# Virtual reality-based therapy associated with blood flow restriction in older adults: A proposal for integration of techniques

**DOI:** 10.3389/fphys.2022.958823

**Published:** 2022-08-19

**Authors:** Silas de Oliveira Damasceno, Eduardo Pizzo Junior, Leonardo Kesrouani Lemos, Taíse Mendes Biral, Allysiê Priscilla de Souza Cavina, Franciele Marques Vanderlei

**Affiliations:** ^1^ Postgraduate Program in Movement Sciences, Universidade Estadual Paulista (FCT/UNESP), Presidente Prudente, SP, Brazil; ^2^ Postgraduate Program in Physiotherapy, Universidade Estadual Paulista (FCT/UNESP), Presidente Prudente, SP, Brazil; ^3^ Department of Physiotherapy, Universidade Estadual Paulista (FCT/UNESP), Presidente Prudente, SP, Brazil

**Keywords:** virtual reality, exergame, kaatsu training, occlusion training, older adults

## Introduction

Although traditional training methods are more common in clinical practice, information on new forms of treatment through exercise are growing in the literature, such as virtual reality-based therapy (VRT) (Amorim et al., 2018) and blood flow restriction (BFR) (Clarkson et al., 2017). Although there are well-conducted studies with both therapies in isolation (Cook et al., 2013; Centner et al., 2019a) and in diverse populations (Takada et al., 2012; Ferreira et al., 2019), there are no studies in the literature for any populations combining these techniques. Therefore, research involving these two training methods is necessary to promote knowledge of the physiological mechanisms inherent in the combination of the techniques, especially for the older population.

It is known that aging is a natural process, characterized by systemic physiological alterations in the individual (Dziechciaż and Filip, 2014), however, it has already been established that the practice of physical exercise is an important component to minimize the damage caused by aging (Roberts et al., 2017). On the other hand, there should be a systematic approach to prescribing physical exercise for this population, as some older adults may not tolerate high training loads and intensities, considering VRT and BFR can make a new low-intensity training modality viable and with the same impact as high-intensity training for older adults (Lee et al., 2017).

In this sense, BRF, a technique developed in the 90 s and which has seen scientific growth in recent years. This method is also synonymous with Kaatsu (Kaatsu Global, Inc.), vascular occlusion training, and hypoxic training. It uses a pneumatic cuff inflation or cuff to partially blood flow restriction while occluding venous flow until cuff pressure is released. Furthermore, in this method, training loads are generally lower, which offers the professional a means of mitigating the effects of aging on muscle function (Loenneke et al., 2012; Dankel et al, 2017). It is believed that adaptations in muscle function with the use of BFR may occur due to the intramuscular hypoxic environment and metabolic stress, inducing, respectively, a progressive increase in the recruitment of motor units of fast-twitch fibers and an activation of mTOR and MAPK myogenic signaling pathways, thus contributing to increases in muscle strength and hypertrophy (Centner et al., 2019b).

Both actions are believed to induce changes in gene expression and anabolic signaling (Gundermann et al., 2014; Ellefsen et al., 2015) and when associated with low intensity exercises, BRF has been shown to be effective for the older population in different contexts and in different outcomes, mainly muscle strength and hypertrophy (Ellefsen et al., 2015; Kim et al., 2016). In addition, low-intensity exercise associated with BRF showed similar benefits to high-intensity training (Kim et al., 2016). Therefore, this technique could be effective when high loads are not possible or desirable in a training program for older adults.

VRT is a technique that uses exergames or videogames, through interactive games, promoting an interface between an operational processor and the user, creating a safe, enriched, and challenging environment (Costa et al., 2019). This practice has been widely used in recent years, as it has a motivational aspect often not achieved in traditional therapy, which encourages the individual to perform physical exercise (Itakussu et al., 2015). The benefits for the older population are improved balance, mobility, muscle strength, cognition, and others (Staiano and Flynn, 2014).

Thus, considering the positive effects of BFR in the face of different outcomes in older adults and its contribution, above all, to the increase in strength and muscle mass, and that VRT can provide improvement in skills such as balance, coordination, muscle strength, and cognition, the association of these techniques appears as a viable therapy for the adherence of older adults to rehabilitation programs, as well as in situations where high intensity exercises are not recommended for older adults. However, due to the lack of studies in older adults who used these techniques together, future studies are needed to confirm or refute the benefits of this combination. Therefore, this opinion article points to an initial perspective on a possible association between VRT and BFR, describing the characteristics of these techniques to serve as inspiration for future studies.

### Blood flow restriction

For the older population, BFR has been shown to be effective, especially when this population group presents limitations to performing high-intensity exercises, such as health conditions or musculoskeletal injuries (Yasuda et al., 2014). The benefits of this application are already documented for older adults who can benefit from increased muscle strength, and improved levels of muscle mass, functional performance, and bone health (Cardoso et al., 2018).

BRF has been shown to have a positive effect on the muscle strength of older adults, in which a systematic review with meta-analysis, including 11 studies, showed that BRF associated with low intensity exercises led to an increase in muscle strength when compared to the low intensity group without BRF. On the other hand, the group that used high intensity training demonstrated better muscle strength when compared to the low intensity training group associated with BRF (Centner et al., 2019b).

A systematic review that included 30 studies and assessed the effects of BFR on hypertrophy in older adults, identified 20 studies that evaluated this outcome and, of these, 15 studies, showed improvements through an increase in skeletal muscle (Baker et al., 2020). In this same review, the authors found that most of the included studies on physical performance using BFR, analyzed by the timed up and go test and sit and stand test, provided better functional levels in the older population (Baker et al., 2020).

The main intrinsic adversity of aging is sarcopenia and a consequent decrease in strength (Montero-Fernández and Serra-Rexach, 2013). This decline in muscle mass and strength substantially reduces the autonomy of older adults, negatively impacting the performance of activities of daily living (Steffl et al., 2017). Physical exercise has shown great importance to prevent sarcopenia (Mello et al., 2019) and its association with BFR could be a preventive alternative for this population.

Another important point to be discussed is that BFR training showed a significant improvement in bone health markers when compared to the group that did not exercise, probably because BFR promotes the secretory function of endothelial cells, facilitating bone remodeling (McCarthy, 2006), as pointed out in the study of Karabulut et al. (2011).

Although all therapies present potential risks, BFR has been shown to present a low risk of injury. Despite this, some points must be presented: due to the occlusive character provided by the cuff, possible effects on circulation can be found during and after the intervention (Nakajima et al., 2006). Pain, paresthesia, and discomfort may occur due to training (Nakajima et al., 2006), therefore, the application of this technique should be discussed from a safety perspective. Despite the risk of muscle injury and coagulation during the application of this technique being well highlighted, a systematic review pointed out that when adequate application occurs, the risk of endothelial injury does not appear to present high risks (Nascimento et al., 2020).

### Virtual reality-based therapy

VRT is a widespread therapeutic method which includes interaction between the user and an artificial operational base, simulating activities in real time (Laufer et al., 2014). Its applicability has low cost and, therefore, it is feasible in clinical practice, enabling clinical implications in rehabilitation programs for older adults, because it promotes improvements in motor and sensory skills responsible for maintaining independence (Donath et al., 2016).

The VRT can be immersive, when the older adults is transported through application of multisensory devices that capture movements, so that the experience presents a feeling of being inside the virtual world, and non-immersive when the older adult is partially transported to the virtual environment using a console, monitor, joystick, and others to manipulate the virtual environment (Holden and Dyar, 2002).

During VRT, it is necessary that the older adults make multidirectional changes in their center of gravity in a fast and controlled way, associated with cognitive demand, agility, dual task, monitoring of the environment, and selection of visual and auditory stimulus (Gomes et al., 2018). Thus, VRT should be prescribed with caution, as it can represent a risk for older adults if the virtual exercise is not well selected (Gomes et al., 2018). However, a controlled clinical trial demonstrated that the use of the Nintendo Wii Fit Plus video game in frail and pre-frail older adults was feasible, acceptable and safe, in addition to improving postural control and gait in these older adults (Gomes et al., 2018).

The use of VRT is a very important tool in triggering real-time feedback on the performance of older adults, as it provides constant sound and visual stimuli during practice, serving as immediate support that in the long-term favors motor learning (Tsang and Fu, 2016). In this sense, during VRT older adults have freedom to interact with the games, which directly impacts on the simultaneous motor and cognitive training in a motivational and pleasant environment, but with a great rehabilitation character (Bruin et al., 2010).

Although the use of VRT is indicated under professional supervision, a study which evaluated this therapy without supervision with older adults showed that the intervention group presented improvements in hip muscle strength and body balance when compared to the control group who maintained their usual routine. In addition, the authors stated that VRT can be a useful technique to improve the physical functioning of older adults, even outside of a supervisory environment, however, the findings emphasize the need to verify the safety and viability of the application when unsupervised (Kim et al., 2013).

### Virtual reality-based therapy associated with blood flow restriction

VRT is widely used as training for older adults and is widely accepted by this public, as the games are challenging, and provide improvements in balance, posture, mobility, functionality, and motivation and when performed in groups or in pairs, VRT promotes social interaction and a healthy competitiveness (Treml et al., 2013). Furthermore, VRT is related to better rates of therapeutic adherence and well-being in older adults, as video games generate greater attention and focus for solving the task, which promotes improvement in physical performance and adaptability to the task (Meneghini et al., 2016).

Still on the use of VRT, the therapist must pay attention to the games that will be used, so that they promote, among other things, adequate weight transfer, and cyclical, rapid, and coordinated movements, which favor cognitive work, dual tasks, and quick decision making, that is, games in a virtual environment must be adequate for stimulus efficiency, respecting the clinical condition of the older adult and especially considering the risks of this intervention (Skjæret et al., 2016).

In the same sense, the benefits of BFR occur both in its use alone or associated with another type of exercise (Scott et al., 2015). When used alone, BFR attenuates the reduction in muscle mass and strength, when associated with cycling or walking, BFR promotes a moderate increase in and maintenance of muscle mass and strength, and when associated with low intensity resistance exercise, BFR presents a substantial increase in muscle mass and strength (Scott et al., 2015).

For better application of BFR, an important point that must be considered is the width of the cuff used, because despite the literature showing variations in cuff sizes from 3 to 18 cm, the current recommendation points out that different sizes can be used, as long as the cuff occlusion pressure is adjusted to 40–80% of the total occlusion pressure at rest (Patterson et al., 2019).

In addition, it should be considered for discussion, that some precautions must be taken when using BFR. It is known that, for better results of the association of BFR with resistance or aerobic exercise, frequencies of two to three sessions a week are recommended when considering application for over 3 weeks and one or two sessions per day considering application over up to 3 weeks. In addition, the application time of using the BFR is five to 10 min per exercise (Patterson et al., 2019).

If the recommended care is taken, BFR together with VRT could become a viable training method for the older population, especially sarcopenic older adults, as it will maximize the gains generated by the therapies individually. BFR, mainly for the gain in strength and hypertrophy and VRT for the gain in balance, mobility, and cognition, could, together, become an innovative training tool that guarantees greater adhesion of older adults, however, future investigations need to test this hypothesis.

### Final considerations

The current opinion article presented the advantages of integrating VRT with BFR, the first to promote gains in balance, cognition, and postural control and the second to improve strength and hypertrophy in older adults. In view of this, the main objective of the current work was to present the scientific community with an intervention based on the integration of techniques and that, in view of the above, generates scientific debate in view of the current scenario of training for older adults.
